# Insular Connectivity Is Associated With Self-Appraisal of Cognitive Function After a Concussion

**DOI:** 10.3389/fneur.2021.653442

**Published:** 2021-05-21

**Authors:** Nathan W. Churchill, Michael G. Hutchison, Simon J. Graham, Tom A. Schweizer

**Affiliations:** ^1^Keenan Research Centre for Biomedical Science of St. Michael's Hospital, Toronto, ON, Canada; ^2^Neuroscience Research Program, St. Michael's Hospital, Toronto, ON, Canada; ^3^Faculty of Kinesiology and Physical Education, University of Toronto, Toronto, ON, Canada; ^4^Department of Medical Biophysics, University of Toronto, Toronto, ON, Canada; ^5^Physical Sciences Platform, Sunnybrook Health Sciences Centre, Sunnybrook Research Institute, Toronto, ON, Canada; ^6^Faculty of Medicine (Neurosurgery), University of Toronto, Toronto, ON, Canada; ^7^The Institute of Biomaterials and Biomedical Engineering (IBBME) at the University of Toronto, Toronto, ON, Canada

**Keywords:** concussion, mild TBI, self-appraisal, insula, fMRI, DTI

## Abstract

Concussion is associated with acute cognitive impairments, with declines in processing speed and reaction time being common. In the clinical setting, these issues are identified *via* symptom assessments and neurocognitive test (NCT) batteries. Practice guidelines recommend integrating both symptoms and NCTs into clinical decision-making, but correlations between these measures are often poor. This suggests that many patients experience difficulties in the self-appraisal of cognitive issues. It is presently unclear what neural mechanisms give rise to appraisal mismatch after a concussion. One promising target is the insula, which regulates aspects of cognition, particularly interoception and self-monitoring. The present study tested the hypothesis that appraisal mismatch is due to altered functional connectivity of the insula to frontal and midline structures, with hypo-connectivity leading to under-reporting of cognitive issues and hyper-connectivity leading to over-reporting. Data were collected from 59 acutely concussed individuals and 136 normative controls, including symptom assessments, NCTs and magnetic resonance imaging (MRI) data. Analysis of resting-state functional MRI supported the hypothesis, identifying insular networks that were associated with appraisal mismatch in concussed athletes that included frontal, sensorimotor, and cingulate connections. Subsequent analysis of diffusion tensor imaging also determined that symptom over-reporting was associated with reduced fractional anisotropy and increased mean diffusivity of posterior white matter. These findings provide new insights into the mechanisms of cognitive appraisal mismatch after a concussion. They are of particular interest given the central role of symptom assessments in the diagnosis and clinical management of concussion.

## Introduction

Concussion is a form of mild traumatic brain injury (mTBI) that typically presents without overt neuroanatomical lesions. Nevertheless, injury is associated with a range of symptoms, as well as measurable declines in processing speed, reaction time, memory, and executive function (1). Symptom assessment remains the cornerstone of clinical management in concussion, and includes patient self-evaluation of cognitive issues (2). It has been observed, however, that correlations between symptom assessments and performance on objective neurocognitive tests (NCTs) are often weak (3–5). These findings suggest a misalignment between self-appraised cognitive abilities and performance on NCTs (i.e., “appraisal mismatch”). This poses a challenge, as guidelines highlight the importance of integrating symptom and NCT data during clinical decision-making (1). It is therefore of importance to better understand the neural mechanisms of appraisal mismatch among concussed individuals.

The insula is likely to play an important role in self-appraisal after a concussion. This region is an integration hub that exhibits dense structural and functional connections throughout the brain and it is reliably activated during cognitive tasks, particularly those involving interoception and self-monitoring (6, 7). At the network level, the insula is involved in the integration of salient stimuli and in mediating the balance between networks subserving internally-directed and externally-directed modes of cognition (8, 9). Thus, the insula plays a key role at the nexus of cognition, symptoms and self-appraisal. Its importance in concussion outcome is also supported by prior neuroimaging studies, where insular functional connectivity and cerebral blood flow were shown to correlate with the severity of self-reported symptoms and with performance on NCTs (10–12). Altered insular connectivity, both functional and structural, may therefore play a role in the appraisal mismatch frequently seen after a concussion.

In anticipating the effects of concussion on self-appraisal and insular connectivity, we may consider other neurological conditions, where more profound deficits of self-appraisal are commonly observed. In cohorts with mild cognitive impairment (MCI) and Alzheimer's Disease (AD), anosognosia, or a lack of awareness for functional and cognitive deficits, has been correlated with more rapid disease progression (13, 14) and with greater prefrontal structural and functional abnormalities (15–18). There is a smaller body of literature examining these issues in TBI, where deficits of self-appraisal do not typically rise to the level of anosognosia but are nevertheless correlated with worse functional outcomes (19–21). In this context, cerebral lesions have been linked to impaired self-awareness (22) and increased prefrontal activity during a self-appraisal task has been linked to a greater awareness of deficits (23). Based on this body of literature, it is expected that post-concussion appraisal mismatch is associated with altered insular connectivity to prefrontal regions and to midline regions, including the cingulate cortex (17).

Studies of self-appraisal in clinical cohorts have historically focused on the under-reporting of cognitive deficits. The under-reporting of cognitive symptoms relative to NCT performance is a concern in concussion, particularly in the sport context where this may lead to premature return to play, putting athletes at risk for re-injury (24, 25). However, there is also concern about the over-reporting of cognitive symptoms relative to NCT performance, given the complex issues surrounding persistent post-concussion symptoms, which may occur without substantial NCT deficits (26, 27). These issues are important to investigate in concussion, where injury processes are subtle and distinct from those previously studied, i.e., in cohorts of patients with more severe TBI or with neurodegenerative disease. Supporting this assertion, it has been noted that the neural substrates of altered self-appraisal likely depend on the mechanisms of neurological injury and the measures by which self-appraisal is defined (28).

The present study examined a cohort of 59 concussed athletes, imaged acutely using resting-state functional magnetic resonance imaging (rs-fMRI), along with a substantial normative cohort of 136 athletic controls without recent concussion. Cognition was evaluated in terms of speed of task performance on an NCT battery, as slowing of response time is one of the most consistently observed deficits in NCTs after concussion (29–32). This likely reflects a general reduction in speed of information processing (33), which has a common neural substrate across different tasks (34). Potential discrepancies between relative NCT performance and the relative severity of self-reported cognitive symptoms (i.e., “appraisal mismatch”) were also assessed. A flexible multivariate approach was then used to test for associations between appraisal mismatch and functional connectivity of the insular cortex. Given role of the insula in self-monitoring, it was hypothesized that reduced connectivity to prefrontal and midline regions corresponds to under-estimation of NCT impairments, whereas enhanced connectivity corresponds to an over-estimation of NCT impairments. In addition, diffusion tensor imaging (DTI) was used to examine white matter microstructure among concussed athletes and controls. It was hypothesized that the functional correlates of appraisal mismatch show corresponding patterns of microstructural disturbances among concussed individuals, as captured by DTI.

## Materials and Methods

### Study Participants

Fifty-nine (59) athletes were recruited consecutively from university-level teams at a single institution through the sports medicine clinic, following diagnosis of sport-related concussion (see Supplementary Material 1 for sport numbers). Diagnosis was determined by staff physician following sustained direct or indirect contact to the head, with assessment of clinical features as per Concussion in Sport Group guidelines (35) and neurologic assessment including examination of cranial nerves, gait, balance and gross motor function. Imaging of was conducted acutely within the 1st week of injury [median and interquartile range (IQR): 5 (2, 7) days]. One hundred and thirty-six (136) control athletes were also recruited consecutively at the start of their competitive season, and were required to be fully recovered clinically from any prior concussions. Both cohorts included athletes with and without history of concussion (HOC), to ensure the samples are representative of normal athlete demographic variability. None of the athletes recruited for the study had a history of neurological or psychiatric diseases or sensory/motor impairments. Within the cohorts, 56/59 (95%) concussed and 127/136 (93%) controls were right-handed; the 12 left-handed athletes were retained after verifying the individual functional connectivity patterns were not outliers relative to the right-hand sample (*p* > 0.101 for all subjects, using the procedure described in Magnetic Resonance Imaging below) and that their exclusion did not significantly alter parameter estimates of the main study analyses, based on bootstrapped comparisons of model regression coefficients with/without left handed athletes included (*p* > 0.898, for all tests).

For all participants, computerized neurocognitive testing was administered at the time of imaging using the C3 Logix platform, which is designed for longitudinal assessment of domains typically affected by concussive blows (e.g., reaction time, processing speed, memory, and visuospatial function), with good test-retest reliability and validity (36, 37). Subtests were presented in a fixed order for all participants within a single session, but with randomization between sessions. The present study focused on four subtests of varying complexity that capture speed of task performance, including Simple reaction time (SRT), Choice reaction time (CRT), Trail-Making Test A (TMT-A), and Trail-Making Test B (TMT-B). Mean reaction time was evaluated for the SRT and CRT tasks and total completion time was evaluated for the TMT-A and TMT-B tasks. The C3 Logix platform also includes the components of the Sport Concussion Assessment Tool 5 (SCAT5) (2), which is a standardized tool that is widely used to assess symptoms neurological function and balance after concussion, with good reliability and validity (38–40). The SCAT5 is a 22-item scale listing typical post-concussion symptoms, with each item receiving a 7-point Likert scale rating. The present study obtained a cognitive symptom severity score (SYM) by summing the Likert ratings for the six symptom scale items that probe cognition (“feeling slowed down,” “feeling 'in a fog',” “don't feel right,” “difficulty concentrating,” “difficulty remembering,” and “confusion”).

Recruitment and data collection were conducted between August 2016 and March 2019 in accordance with the Canadian Tri-Council Policy Statement 2 and approval of University of Toronto and St. Michael's Hospital research ethics boards, with participants giving free and informed consent. The datasets analyzed for this study can be found in the *figshare* repository at https://figshare.com/s/d758e5e696332f8b3edf.

### Magnetic Resonance Imaging

Athletes were imaged using a 3 Tesla MRI system (Magnetom Skyra) with a standard 20-channel head coil. Structural imaging included: three-dimensional T1-weighted Magnetization Prepared Rapid Acquisition Gradient Echo imaging [MPRAGE: inversion time (TI)/echo time (TE)/repetition time (TR) = 1,090/3.55/2,300 ms, flip angle (θ) = 8°, 192 sagittal slices with field of view (FOV) = 240 × 240 mm, 256 × 256 pixel matrix, 0.9 mm slice thickness, 0.9 × 0.9 mm in-plane resolution, with bandwidth (BW) = 200 Hertz per pixel (Hz/px)], fluid attenuated inversion recovery imaging (FLAIR: TI/TE/TR = 1,800/387/5,000 ms, 160 sagittal slices with FOV = 230 × 230 mm, 512 × 512 matrix, 0.9 mm slice thickness, 0.4 × 0.4 mm in-plane resolution, BW = 751 Hz/px) and susceptibility-weighted imaging (SWI: TE/TR = 20/28 ms, θ = 15°, 112 axial slices with FOV = 193 × 220 mm, 336 × 384 matrix, 1.2 mm slice thickness, 0.6 × 0.6 mm in-plane resolution, BW = 120 Hz/px). The MPRAGE, FLAIR and SWI scans were inspected by an MRI technologist during imaging and later reviewed by a neuroradiologist, with clinical reporting if abnormalities were identified. No abnormalities (white matter hyper-intensities, contusions, micro-hemorrhage, or statistical outliers) were found for the athletes in this study.

#### Functional MRI (fMRI)

Resting-state fMRI was acquired *via* multi-slice T2^*^-weighted echo planar imaging (EPI: TE/TR =30/2,000 ms, θ = 70°, 32 oblique-axial slices with 200 × 200 mm FOV, 64 × 64 matrix, 4.0 mm slice thickness with 0.5 mm gap, 3.125 × 3.125 mm in-plane, 2,298 Hz/px BW), producing a series of 195 images for each slice. During acquisition, athletes were instructed to lie still with their eyes closed and to not focus on anything. Data processing was performed as described in prior publications (12, 41, 42) and was based on Analysis of Functional Neuroimages (AFNI; afni.nimh.nih.gov), the FMRIB Software Library (FSL; https://fsl.fmrib.ox.ac.uk) and customized algorithms. After discarding the first four images in each time series to allow magnetization to attain equilibrium, we performed rigid-body motion correction using AFNI *3dvolreg*, removal and interpolation of outlier volumes with SPIKECOR (nitrc.org/projects/spikecor), slice-timing correction *via* AFNI *3dTshift*, spatial smoothing with a 6 mm Full Width at Half Maximum (FWHM) isotropic 3D Gaussian kernel using AFNI *3dmerge*, along with regression of motion parameters and linear-quadratic trends. For motion parameter regression, principal component analysis was performed on the six rigid-body parameters, and the first two components used as regressors. To control for physiological noise, the PHYCAA+ algorithm (nitrc.org/projects/phycaa_plus) was used to down-weight non-neural signal, using an approach that rescales voxel variance based on the proportion of high-frequency BOLD power content seen above 0.10 Hz (43). This was followed by regression of signal from white matter (WM) and cerebrospinal fluid (CSF). The WM and CSF regressions were performed following spatial normalization, described below.

The fMRI data were then co-registered to a common anatomical template space. For each athlete, we used FSL *flirt* to obtain the rigid-body transform of their mean fMRI image to their T1-weighted scan and the affine transformation of their T1 image to the MNI152 template. The net transform was then applied to the fMRI images, with resampling at 3 mm isotropic voxel resolution. To remove WM and CSF signal, subject T1-weighted images were segmented and co-registered to the MNI152 template using the FSL *fslvbm* protocol and averaged to produce cohort-specific probabilistic tissue templates. These templates were resampled into 3 mm isotropic voxel resolution and smoothed with a 6 mm FWHM isotropic 3D Gaussian kernel. Masks were then obtained of voxels in the 95th percentile, for both WM and CSF maps. After, two WM time series were obtained by averaging voxels within cerebral and brainstem white matter, and two CSF time series were obtained by averaging voxels within left and right lateral ventricles. The four physiological time series were regressed from each dataset. To reduce computational burden and improve the stability of BOLD measures, we parcellated the data using the Brainnetome Atlas (BNA), which consists of 246 cortical and subcortical parcels, including 12 insular subregions (44). We then obtained mean seed time series by averaging over all voxels within a parcel, generating 246 BOLD time series. The Pearson correlation was afterwards calculated between each pair of seed time series time series, obtaining a 246 × 246 functional connectivity matrix for each study participant.

#### Diffusion Tensor Imaging

A diffusion tensor imaging (DTI) protocol was performed (66 axial slices with FOV = 240 × 240 mm, 120 × 120 matrix, 2.0 mm slice thickness, 2.0 × 2.0 in-plane resolution, BW = 1,736 Hz/Px), consisting of 30 diffusion-weighting directions (TE/TR = 83/7,800 ms, b = 700 s/mm^2^, with nine b0 scans). The data were processed using FSL utilities and custom software. The FSL *eddy* protocol was used to perform simultaneous correction of eddy currents and rigid-body head motion, FSL *bet* was used to mask out non-brain voxels, and FSL *dtifit* used to calculate voxel-wise measures of fractional anisotropy (FA) and mean diffusivity (MD).

Co-registration of DTI maps to a common template was obtained using Diffusion Tensor Imaging ToolKit (DTI-TK; http://dti-tk.sourceforge.net/) with default parameter settings. The IXI Aging DTI Template 3.0 was used as an initial reference, and an independent randomly-selected, demographically matched subgroup of 60 athletic controls was used to generate an athlete template [mean age 20.2 ± 1.7, 31/60 female (52%) and 26/60 with HOC (43%)], chosen to reduce computational burden while ensuring a large enough sample to be representative of normal demographic variability. For this group, a bootstrapped template was obtained with *dti_template_bootstrap*, affine alignment and template updating was done using *dti_affine_population* (three iterations), then diffeomorphic alignment and template updating was done with *dti_diffeomorphic_population* (three iterations). The transform from athletic template to MNI space was afterwards obtained using the IIT Human Brain Atlas' mean tensor template by sequentially applying rigid (*dti_rigid_reg*), affine (*dti_affine_reg*), and diffeomorphic (*dti_diffeomorphic_reg*) registration steps. For all athletes in this study, transforms to the athlete group template were then obtained by sequentially applying rigid (*dti_rigid_reg*), affine (*dti_affine_reg*), and diffeomorphic (*dti_diffeomorphic_reg*) steps. After, the net transforms into MNI space were computed using *dfRightComposeAffine* and were applied to DTI parameter maps *via deformationScalarVolume*. During registration, images were resampled to 3 mm isotropic voxel resolution, and a 6 mm FWHM 3D Gaussian smoothing kernel applied to reduce spatial noise. Voxel-wise analysis was performed within a mask of probable white matter regions, i.e., including all brain voxels where mean FA > 0.30 for the template subgroup, with manual segmentation and exclusion of brain stem areas with substantial field inhomogeneity.

#### Outlier Detection

As this study focused on modeling multivariate covariance relationships, which are highly sensitive to extreme data points, all imaging data were tested for outliers using a multivariate approach (see Supplementary Material 2 for details). For fMRI data, one (1) concussed outlier was identified. For DTI data, two (2) control participants and one (1) concussed participant were identified as outliers. In addition, two (2) concussed athletes had missing DTI data and were not included in the DTI-based analyses.

### Modeling Brain-Behavior Relationships

The BNA parcellation subdivides the insula into 12 subregions: hypergranular (G), dorsal granular (dIg), dorsal dysgranular (dId), dorsal agranular (dIa) ventral dysgranular/granular (vId/vIg), and ventral agranular (vIa), with left and right hemispheric subdivisions. Because the different insular subregions play a diverse set of roles (7) and it is *a priori* unclear which subregions are most relevant to self-appraisal, a flexible multivariate approach called N-way partial least squares (NPLS) (45) was used for analysis. The NPLS method has been implemented in neuroimaging studies of concussion and identifies distributed brain patterns that show covariation with clinical indices (11, 12, 46). It is an extension of the widely-used partial least squares (PLS) algorithm (47, 48), which identifies latent covariance relationships between paired multivariate datasets, and finds stable solutions in the presence of high-dimensional imaging data and often multicollinear behavioral data.

For standard neuroimaging PLS, datasets typically consist of (*V* × 1) vectors of brain values ***x***_*s*_ and (*B* × 1) vectors of behavioral values ***y***_*s*_, obtained for each subject *s* = 1…*S*. The PLS model quantifies shared information between these datasets by extracting *k* = 1…*K* latent variable pairs from ***x*** and ***y***, denoted $\left(c_{k}^{x},c_{k}^{y}\right)$. For the *k*^th^ latent variable pair, the model identifies weight vectors $w_{k}^{x}$ and $w_{k}^{y}$ producing latent variables $c_{k\left(s\right)}^{x}={x_{s}}^{T}w_{k}^{x}$ and $c_{k\left(s\right)}^{y}={y_{s}}^{T}w_{k}^{y}$ that maximize the covariance expression:

(1) $$\begin{array}{l} cov\left(c_{k}^{x},c_{k}^{y}\right)=cov\left({w_{k}^{x}}^{T}x_{s},\;{w_{k}^{y}}^{T}y_{s}\right),\text{such that}||w_{k}^{x}||=||w_{k}^{y}||=1 \end{array}$$

where $\left\{w_{k}^{x},w_{k}^{y}\right\}$ are orthogonal to the previous *k*−1 weight vectors. For this model, expression of the multivariate brain pattern $w_{k}^{x}$ is associated with pattern of behavioral response $w_{k}^{y}$, with strength of the covariance relationship quantified by $cov\left(c_{k}^{x},c_{k}^{y}\;\right)$.

For neuroimaging NPLS, the model is extended to reflect the more general scenario where the imaging dataset consists of (*V*_1_ × *V*_2_) matrices of brain values *X*_*s*_ for each subject, as in the case of functional connectivity matrices. For the ***k***^th^ latent variable pair, the model identifies weight vectors $w_{k}^{x1}$, $w_{k}^{x2}$, and $w_{k}^{y}$ that produce latent variables $c_{k\left(s\right)}^{x}={w_{k}^{x1}}^{T}X_{s}w_{k}^{x2}$ and $c_{k\left(s\right)}^{y}={y_{s}}^{T}w_{k}^{y}$ that maximize the covariance expression:

(2) $$\begin{array}{l} cov\left(c_{k}^{x},c_{k}^{y}\right)=cov\left({w_{k}^{x1}}^{T}X_{s}w_{k}^{x2},\;{y_{s}}^{T}w_{k}^{y}\right),\\ \text{ such that}||w_{k}^{x1}||=||w_{k}^{x2}||=||w_{k}^{y}||=1 \end{array}$$

where model parameters are optimized using iterative updating (45). For the present study, subject data consists of (12 × 246) submatrices ***X***_*s*_ of connectivity values between the 12 insular subregions of the BNA and the full set of 246 brain parcels in the BNA. In this case, NPLS identifies (12 × 1) insular weight vectors $w_{k}^{x1}$ and (246 × 1) full-brain weight vectors $w_{k}^{x2}$ where the “network expression scores” $c_{k}^{x}$ (i.e., the total connectivity between subnetworks) have maximal covariance with clinical indices. This model can be thought of as a kind of flexible seed-based analysis, where NPLS automatically finds the optimal weighting of voxels within the seed region ($w_{k}^{x1}$), along with the brain regions where seed connectivity covaries most strongly with the clinical measure(s) of interest ($w_{k}^{x2}$). Confidence bounds on the NPLS parameter estimates are then obtained using non-parametric resampling approaches, further described in section Analysis of Demographic and Clinical Data below.

### Analysis of Demographic and Clinical Data

Initial analyses compared demographic data between the control and concussed cohort, including mean age, sex (percentage female) and HOC (percentage with prior concussion). The mean difference was estimated for each, with bootstrap resampling (1,000 iterations) to obtain the 95% confidence intervals (95% CIs), bootstrap ratios (BSRs; a z-distributed estimate of standardized effect size, calculated as the mean / standard error), and empirical *p*-values. For the subsets of control and concussed athletes with HOC, similar bootstrap analyses were conducted to test for mean differences in the total number of prior concussions and months since their most recent concussion.

Subsequent bootstrap analyses tested for effects of concussion on the individual C3Logix cognitive subtests (SRT, CRT, TMT-A, and TMT-B). A data-driven composite score was also obtained summarizing overall cognitive performance (COG) by performing Principal Component Analysis (PCA) on scores of the four subtests, for controls and concussed athletes combined. The first component provided the COG summary score, which accounted for greatest overall covariance in performance of cognitive tasks, with larger values denoting slower task performance. Before running PCA, the data were normalized *via* an inverse empirical distribution function and mean centered. Bootstrap resampling (1,000 iterations) was also used to obtain 95%CIs on the PCA parameters. Bootstrap analyses then tested for effects of concussion on both the objective index of cognitive performance COG and subjective assessment of cognitive symptoms SYM. Associations between COG and SYM scores were also examined, along with the demographic factors of age, sex, and HOC using Spearman correlations with bootstrapped 95%CIs and empirical *p*-values.

### Analysis of Functional Connectivity Data

Initial NPLS analyses compared functional connectivity of the insula for athletes with and without concussion. This was done by modeling covariation between the (12 × 246) connectivity submatrices and scalar value *y*_*s*_ coding for group membership (-1 = control, 1 = concussed), which is equivalent to a discriminant PLS model. Statistical inference was conducted by bootstrap resampling on subjects (1,000 iterations) to obtain empirical distributions over the insular saliences *w*^*x*1^ and full-brain saliences *w*^*x*2^. Standardized effect sizes were reported as bootstrap ratios (BSRs), with *p*-values derived from normally-distributed BSRs and thresholding conducted at a False Discovery Rate (FDR) threshold of 0.05. For latent variables with significant saliences, the mean difference in network scores $c_{\left(s\right)}^{x}$ was also reported, along with the bootstrapped 95% CI, BSR and *p*-value.

Associations between insular connectivity, cognition, and symptoms were subsequently assessed for the concussed athlete cohort, based on the summary COG and SYM measures. Both variables were first standardized by applying a normal scores transform over concussed athletes (49), i.e., mapping values to their corresponding quantiles in a standard normal distribution. The new variables z(SYM) and z(COG) measured severity of self-reported and objective cognitive issues respectively, relative to the overall concussed cohort. Orthogonal composite scores z(SYM)+z(COG) and z(SYM)–z(COG) were then computed. The sum of scores z(SYM)+z(COG) reflects overall severity of cognitive issues, both objective and subjective. This is seen by noting that the sum of scores is largest in magnitude when both z(SYM) and z(COG) are large. Conversely, the difference of scores z(SYM) – z(COG) reflects self-appraisal mismatch. Negative values are obtained when athletes have relatively high cognitive impairment z(COG) and endorse relatively low symptoms z(SYM), denoting under-reporting; whereas the opposite relationship, of low z(COG) but high z(SYM), denotes over-reporting. When athletes have “well-calibrated” self-appraisal, z(COG) ≈ z(SYM) and the difference score is near zero. This score fulfills the requirements for assessing accuracy of self-appraisal for cognitive issues, with respect to the overall concussed athlete cohort.

The NPLS analyses were conducted for both composite variables, producing saliences consisting of insular subnetwork *w*^*x*1^ and a full-brain subnetwork *w*^*x*2^, with “network expression scores” $c_{\left(s\right)}^{x}$ that had maximum covariance with the clinical variable of interest. Bootstrapping was again used to obtain BSRs and *p*-values on network saliences, with thresholding at an FDR of 0.05. Afterwards, for latent variables with significant saliences, the composite score was regressed against functional network scores $c_{\left(s\right)}^{x}$, producing coefficient of effect *b* with a bootstrapped 95%CI, BSR and *p*-value. Preliminary analyses found no evidence for effects of z(SYM)+z(COG) on functional connectivity and are therefore not discussed further. Relationships with the difference scores z(SYM) – z(COG) are reported below.

### Analysis of Diffusion-Weighted Data

Analyses also examined the patterns of white matter microstructure that were associated with insular functional networks. For the network expression scores $c_{\left(s\right)}^{x}$ that were related to appraisal mismatch among concussed athletes, additional PLS analyses tested for significant associations with the FA and MD voxel-wise maps. For each DTI parameter, the analyses produced spatial maps *w*^*x*^ of affected white matter, along with latent variable “diffusivity scores” $d_{\left(s\right)}^{x}={w^{x}}^{T}x_{s}$ that had maximal covariance with $c_{\left(s\right)}^{x}$. Bootstrapping was against used to obtain *p*-values and BSRs for the voxel saliences, with thresholding at an FDR of 0.05, and with additional conservative thresholding at a minimum cluster size of 3 to remove singleton clusters and improve interpretability. For latent variables with significant saliences, the network expression scores $c_{\left(s\right)}^{x}$ were then regressed against diffusivity scores $d_{\left(s\right)}^{x}$, producing coefficient of effect *b*, along with a bootstrapped 95%CI, BSR and *p*-value.

## Results

### Demographic and Clinical Data

Participant demographics and clinical data are summarized in Table 1. Both cohorts spanned a similar age range and included a balanced sample of male and female athletes, with and without HOC. Among those with HOC, the concussed athletes did not differ significantly from controls in number of previous injuries, or in time since their last injury. Examining cognitive scores, the acutely concussed athlete group was significantly slower on all tasks, consistent with expectations. Standardized effect sizes, given as BSRs, were generally comparable across all tests, although TMT-B showed the greatest effect of concussion on performance. The composite score COG was obtained from a PCA with positive loadings on all subtests: SRT = 0.475 [95%CI: (0.413, 0.532)], CRT = 0.553 [95%CI: (0.522, 0.589)], TMT-A = 0.442 [95%CI: (0.358, 0.498)], and TMT-B = 0.523 [95%CI: (0.482, 0.557)]. This component accounted for 54.0% of NCT covariance (95%CI: 47.9%, 59.8%) and scores differentiated concussed and control groups with slightly greater sensitivity than individual subtests (i.e., with an increased BSR value), supporting its utility as a summary measure. Examining cognitive symptom score SYM, controls endorsed minimal symptoms, whereas concussed athletes showed consistently higher values and greater inter-individual variability in severity scores. The summary scores COG and SYM show a weakly positive but non-significant Spearman correlation [ρ and 95%CI: 0.214, (−0.066, 0.481), *p* = 0.132]. Neither score was significantly associated with age, sex, or HOC (|ρ| < 0.179 with *p* > 0.184 for all tests).

**Table 1:** Demographic, cognitive, and symptom data for athletes with concussion and controls.

	**Control**	**Concussed**	**Mean difference**	**BSR**	***p*-value**
Age (yr.)	20.3 ± 2.0	20.6 ± 2.1	0.3 (−0.3, 0.9)	0.88	0.399
Female	67/136 (49%)	29/59 (50%)	0 (−15, 16)%	−0.01	0.973
History of concussion (HOC)	55/136 (40%)	28/59 (47%)	7 (−8, 21)%	0.89	0.392
Number of prior concussions	1 (1,2)	2 (1,3)	0 [0, 1]	0.59	0.529
Time since last concussion (mo.)	35 (13, 54)	27 (15, 44)	−6 [−18, 6]	−0.98	0.333
SRT (ms)	262.5 [249.9, 283.1]	271.1 [250.5, 297.8]	11.3 [1.1, 21.1]	2.13	0.025
CRT (ms)	363.0 [337.7, 404.5]	374.0 [349.2, 434.8]	22.9 [3.2, 43.3]	2.20	0.021
TMT-A (s)	16.21 [13.12, 19.36]	18.00 [14.31, 20.93]	1.65 [0.025, 3.54]	1.89	0.047
TMT-B (s)	31.83 [26.79, 38.55]	36.28 [31.03, 43.11]	3.65 [0.91, 6.49]	2.54	0.006
COG (x10^−2^)	−1.16 [−6.65, 4.05]	2.39 [−2.78, 6.87]	3.22 [1.12, 5.36]	3.05	0.002
SYM	0 [0, 1]	6 (2, 16)	8.0 [5.9, 10.1]	7.39	<0.001

*The demographic, cognitive, and symptom data for each group are summarized by the median and interquartile range [Q1, Q3]. Group differences are summarized by the mean and bootstrap 95% confidence interval, with corresponding bootstrap ratio (BSR) and p-value. For athletes with history of concussion (HOC), the number of prior concussions and months since last concussion are also summarized. Cognitive data include reaction times for Simple reaction time (SRT) and Choice reaction time (CRT) tasks, along with completion times for Trail-Making Test A (TMT-A) and Trail-Making Test B (TMT-B). The cognitive composite score (COG) is obtained as the first principal component for the four cognitive tests. The cognitive symptom score (SYM) is obtained by summing scores for all cognitive items in the sport concussion assessment tool 5 (SCAT5)*.

### Functional Connectivity Data

**Figure 1:**
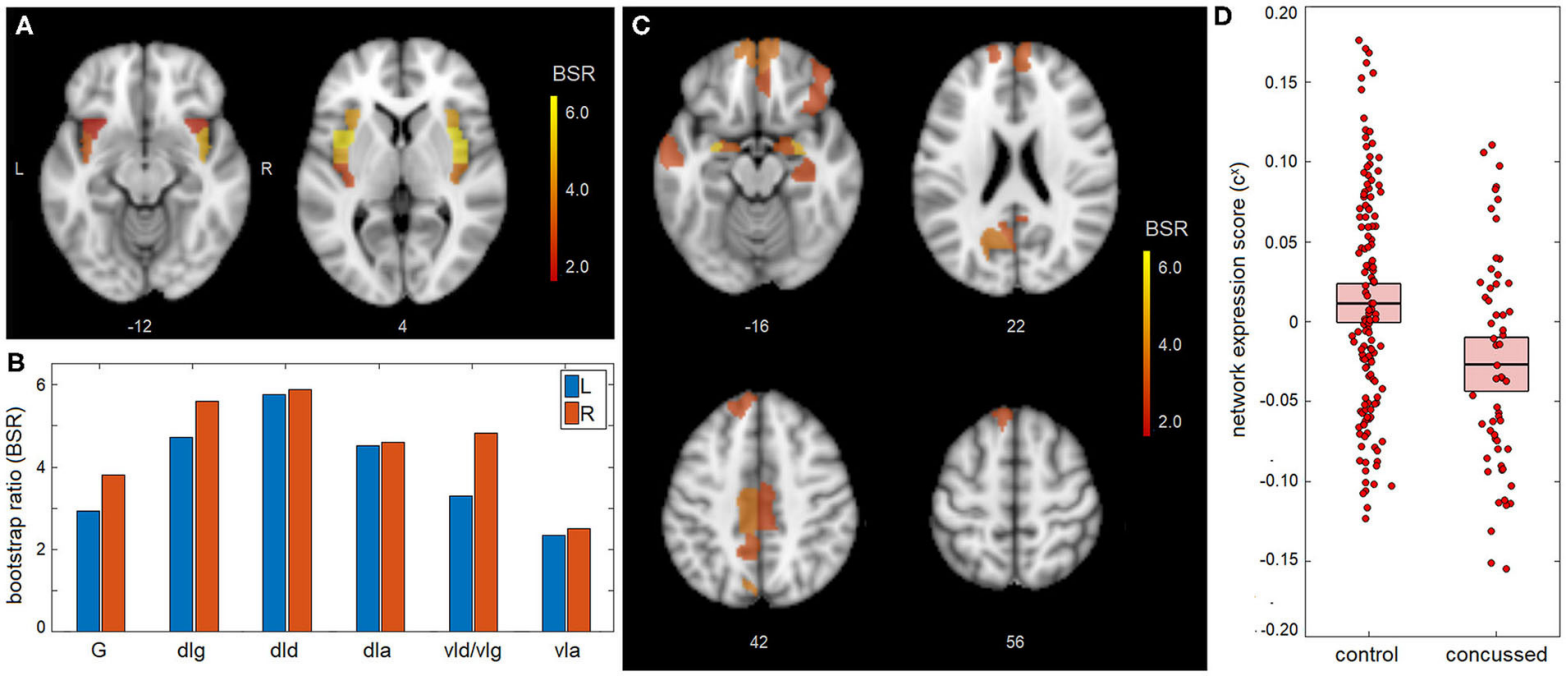
Mean effect of concussion on insular network connectivity. **(A,B)** For the insular subnetwork, salience bootstrap ratios (BSRs) are displayed as a spatial map and as barplot values, ordered posterior-to-anterior. **(C)** For the full-brain subnetwork, salience BSRs are also shown as a spatial map. **(D)** Network expression scores, reflecting insular connectivity to the identified whole-brain network, are plotted for individual control and concussed athletes. The group means are plotted (horizontal black lines), along with the 95%CIs of the mean (boxes). Insular subregions include: hypergranular (G), dorsal granular (dIg), dorsal dysgranular (dId), dorsal agranular (dIa) ventral dysgranular/granular (vId/vIg), and ventral agranular (vIa).

The main effects of concussion on insular functional connectivity are depicted in Figure 1. The NPLS analyses identified a single latent variable with significant saliences. The insular subnetwork had significant weights for all regions (Figure 1A), although the highest values were seen dorsally, particularly in the dorsal dysgranular insula (dId) subregion (Figure 1B). The insular regions showed significant concussion-related alterations in connectivity to a full-brain subnetwork that included the amygdala, but also extended into medial prefrontal regions of the anterior cingulate and orbital gyrus (Figure 1C and summarized in Table 2). The network expression scores are plotted in Figure 1D, reflecting connectivity of the insular regions in Figure 1A to the brain regions in Figure 1C for individual subjects. Relative to controls, the concussed athletes had lower network expression scores and therefore had significantly reduced connectivity between subnetworks [mean and 95%CI: 3.81 × 10^−2^, (1.83, 5.90) × 10^−2^, BSR = 3.70, *p* < 0.001]. For the concussed athletes, connectivity was not significantly associated with composite score z(SYM)+z(COG) [*b* and 95%CI: −0.34 × 10^−2^, (−1.71, 0.84) × 10^−2^, BSR = −0.53, *p* = 0.547], or with composite score z(SYM)-z(COG) [−1.08 × 10^−2^, (−2.24, 0.10) × 10^−2^, BSR = −1.82, *p* = 0.063].

**Table 2:** Brain regions with significant whole-brain salience effects, indicating a network of regions where insular connectivity is associated with main effects of concussion and the effects of appraisal mismatch; the identified regions correspond to the brain maps shown in Figures 1C, 2C, respectively.

		**Brain region**	**Center of mass (MNI)**			**Bootstrap ratio (BSR)**
main effects of concussion	1	Superior frontal gyrus L (A9l)	−11	49	40	3.01
	2	Superior frontal gyrus R (A10m)	8	58	13	3.19
	3	Orbital gyrus R (A14m)	6	47	−7	3.05
	4	Orbital gyrus R (A12/47o)	40	39	−14	2.98
	5	Orbital gyrus L (A11m)	−6	52	−19	3.68
	6	Orbital gyrus R (A11m)	6	57	−16	3.62
	7	Middle temporal gyrus L (aSTS)	−58	−20	−9	3.05
	8	Precuneus L (dmPOS)	−12	−67	25	3.78
	9	Precuneus L (A31)	−6	−55	34	3.16
	10	Cingulate gyrus R (A23d)	4	−37	32	2.93
	11	Cingulate gyrus L (A23c)	−7	−23	41	3.71
	12	Cingulate gyrus R (A23c)	6	−20	40	2.97
	13	Cingulate gyrus L (A32sg)	−4	39	−2	3.55
	14	Amygdala L (mAmyg)	−19	−2	−20	3.35
	15	Amygdala R (mAmyg)	19	−2	−19	3.23
	16	Amygdala L (lAmyg)	−27	−4	−20	5.03
	17	Amygdala R (lAmyg)	28	−3	−20	4.67
	18	Hippocampus R (cHipp)	29	−27	−10	3.01
appraisal mismatch effects	1	Superior frontal gyrus R (A6m)	7	−4	60	3.32
	2	Middle frontal gyrus L (A6vl)	−32	4	55	3.36
	3	Inferior frontal gyrus R (A44op)	42	22	3	3.11
	4	Precentral gyrus R (A4t)	15	−22	71	3.46
	5	Precentral gyrus L (A6cvl)	−49	5	30	3.37
	6	Paracentral lobule L (A1/2/3ll)	−8	−38	58	3.60
	7	Paracentral lobule L (A4ll)	−4	−23	61	3.57
	8	Paracentral lobule R (A4ll)	5	−21	61	4.75
	9	Postcentral gyrus L (A1/2/3ulhf)	−50	−16	43	3.03
	10	Insular gyrus R (vIa)	33	14	−13	3.06
	11	Cingulate gyrus L (A23c)	−7	−23	41	3.96
	12	Cingulate gyrus R (A23c)	6	−20	40	3.64

*Brain regions correspond to brainnetome atlas (BNA) parcels, with coordinates in Montreal Neurological Institute (MNI) space and bootstrap ratio (BSR) values reported*.

*A9l, lateral area 9; A10m, medial area 10; A14m, medial area 14; A12/47o, orbital area 12/47; A11m, medial area 11; aSTS, anterior superior temporal sulcus; dmPOS, dorsomedial parietooccipital sulcus (PEr); A31, area 31 (Lc1); A23d, dorsal area 23; A23c, caudal area 23; A32sg, subgenual area 32; mAmyg, medial amygdala; lAmyg, lateral amygdala; cHipp, caudal hippocampus; A6m, medial area 6; A6vl, ventrolateral area 6; A44op, opercular area 44; A4t, area 4(trunk region); A6cvl, caudal ventrolateral area 6; A1/2/3ll, area1/2/3 (lower limb region); A4ll, area 4(lower limb region); A1/2/3ulhf, area1/2/3(upperlimb; head and face region); vIa, ventral agranular insula*.

**Figure 2:**
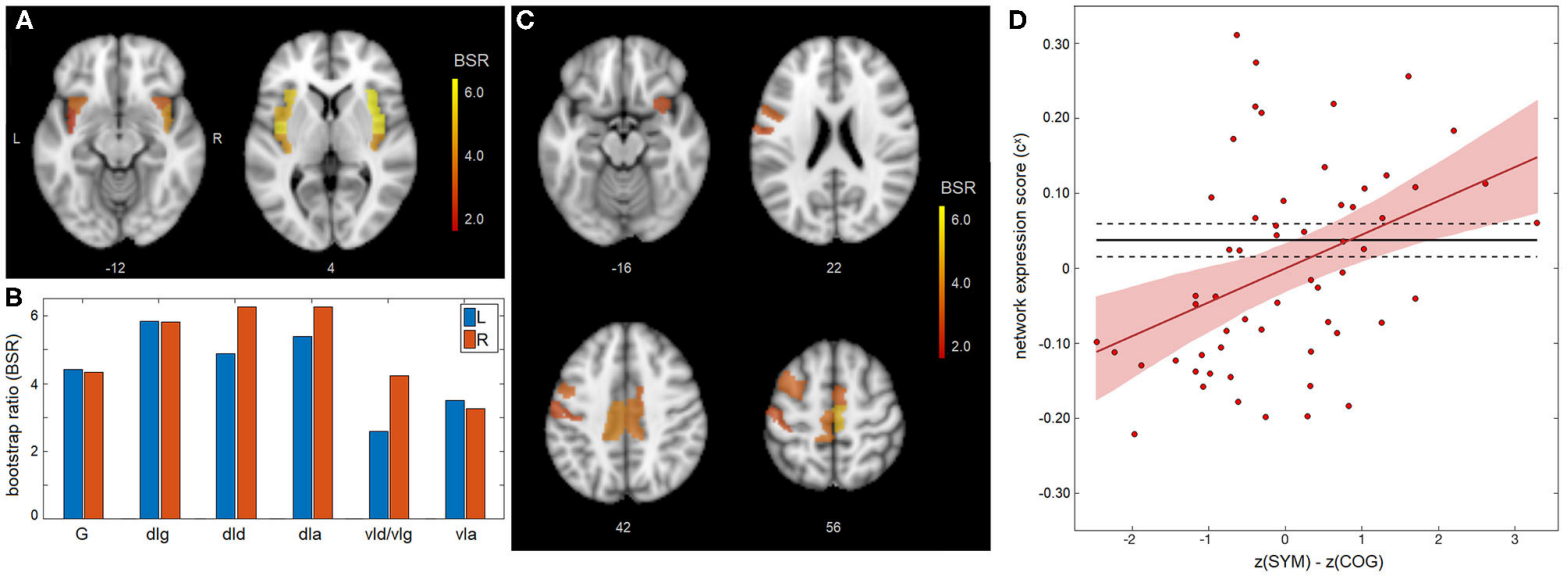
Appraisal mismatch and insular connectivity for the concussed group. **(A,B)** For the insular subnetwork, salience bootstrap ratios (BSRs) are shown as a spatial map and as barplot values, ordered posterior-to-anterior. **(C)** For the full-brain subnetwork, salience BSRs are also shown as a spatial map. **(D)** Network expression scores, reflecting insular connectivity to the identified whole-brain network, are plotted against mismatch scores z(SYM)-z(COG) for individual control athletes. The regression line of best fit is plotted (solid red line), along with its 95%CI (shaded area). The control mean is also plotted (solid black line), along with the 95%CI of the mean (dashed lines). Insular subregions include: hypergranular (G), dorsal granular (dIg), dorsal dysgranular (dId), dorsal agranular (dIa) ventral dysgranular/granular (vId/vIg), and ventral agranular (vIa).

A significant association was found between appraisal mismatch and insular functional connectivity for concussed athletes, shown in Figure 2. As with the main effects, NPLS analyses identified a single latent variable with significant saliences. The insular subnetwork had significant weights for all regions (Figure 2A) and highest values dorsally, now with comparable values in the granular (dIg), dysgranular (dId), and angranular (dIa) subregions (Figure 2B). In this case, however, the insular regions showed significant concussion-related alterations in connectivity to a distinct full-brain subnetwork of mainly prefrontal areas, encompassing superior frontal, middle frontal, and inferior frontal regions. Effects were also seen within the paracentral lobule, sensorimotor cortex and midcingulate, along with a self-connection to the ventral agranular insula (vIa) (Figure 2C and summarized in Table 2). The network expression scores are plotted in Figure 2D, reflecting connectivity of the insular regions in Figure 1A with brain regions in Figure 2C for individual subjects. The analysis identified a modest but significant positive effect of z(SYM) – z(COG) mismatch on connectivity [*b* and 95%CI: 4.51 × 10^−2^, (2.31, 6.58) × 10^−2^, BSR = 4.20, *p* < 0.001], with an *R*^2^ of 0.137 (95%CI: 0.033, 0.312). Thus, athletes who under-estimated cognitive issues [z(SYM)-z(COG) < 0] tended to have reduced connectivity between subnetworks, whereas those who over-estimated cognitive issues [z(SYM)-z(COG) > 0] tended to have elevated connectivity. Interestingly, at the point of z(SYM)-z(COG) = 0 (i.e., well-calibrated appraisal of cognitive issues), the mean connectivity of concussed athletes tended to be lower than that of controls [regression intercept and 95%CI: −3.12 × 10^−2^ (−5.15, −0.99) × 10^−2^, BSR = −2.83, *p* = 0.003].

Secondary analysis of the main effects of concussion found no significant impact of adjusting for age, sex or HOC on the identified relationship brain-behavior relationship (BSR = −0.13, *p* = 0.897). We did identify a significant positive effect of sex on connectivity between subnetworks [*b* and 95%CI: 2.41 × 10^−2^ (0.59, 4.23) × 10^−2^, BSR = −2.53, *p* = 0.008] but no significant effects were identified for age [−0.21 × 10^−2^ (−0.66, 0.25) × 10^−2^, BSR = −0.90, *p* = 0.367] or for HOC [−0.81 × 10^−2^ (−2.86, 1.01) × 10^−2^, BSR = −0.81, *p* = 0.378]. Secondary analysis of the effects of appraisal mismatch similarly found no significant impact of adjusting for age, sex, or HOC on the identified brain-behavior relationship (BSR = 0.12, *p* = 0.905). Sex had a significant negative effect on connectivity between subnetworks [−6.43 × 10^−2^ (−12.61, −0.24) × 10^−2^, BSR = −2.11, *p* = 0.040] but no significant effects were identified for age [−0.43 × 10^−2^ (−2.06, 0.98) × 10^−2^, BSR = −0.55, *p* = 0.539] or for HOC [−3.64 × 10^−2^ (−9.49, 2.89) × 10^−2^, BSR = −1.12, *p* = 0.272].

### Diffusion Weighted Data

**Figure 3:**
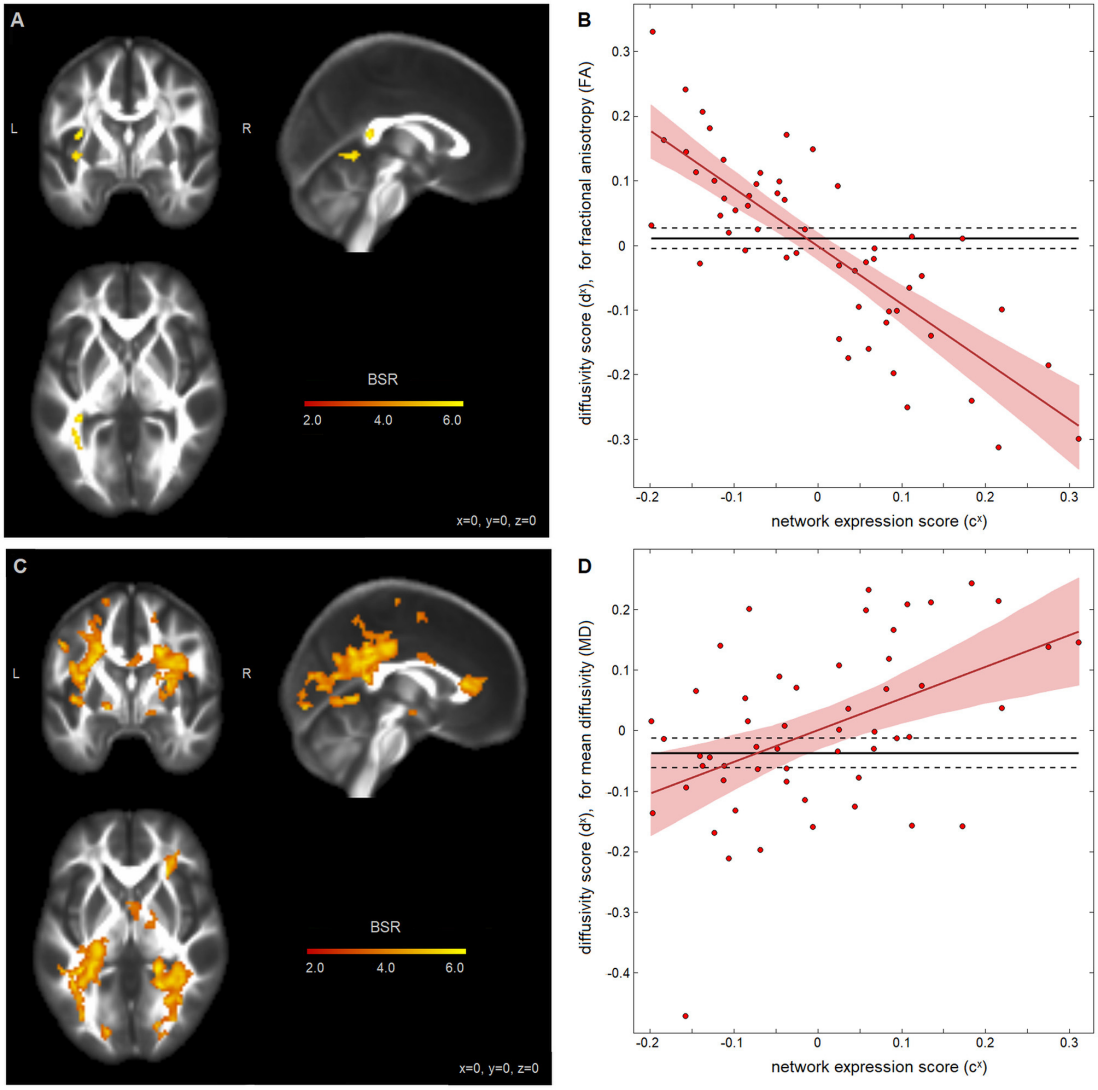
Diffusion-weighted measures and insular network expression scores among concussed athletes. Brain maps show significant effect of fractional anisotropy (FA) in **(A)** and mean diffusivity (MD) in **(C)**, depicted as maximum intensity projections in orthogonal planes [Montreal Neurological Institute (MNI) coordinates: x = 0, y = 0, z = 0]. Scatter plots show the diffusivity scores for **(B)** FA data and **(D)** MD data, plotted against network expression scores for the concussed group, along with the regression line of best fit (solid red line) and its 95%CI (shaded area). The control mean is also plotted (solid black line) with 95%CIs of the mean (dashed lines).

Analysis of the associations between self-appraisal network connectivity and DTI measures revealed significant effects for both FA and MD, displayed in Figure 3, with clusters summarized in Table 3. The FA effects were spatially limited to the left posterior thalamic radiation. In this region, participants with higher self-appraisal network expression scores (reflecting over-endorsement of cognitive issues relative to NCT performance) had reduced FA diffusivity scores [*b* and 95%CI: −0.894, (−1.104, −0.688), BSR = −8.78, *p* < 0.001], with an *R*^2^ of 0.648 [95%CI: (0.491, 0.773)]. The MD effects were more spatially extensive, but also encompassed mainly posterior regions including the thalamic radiations, corona radiata, and longitudinal fasciculi. Within these regions, participants with higher self-appraisal network expression scores had elevated MD diffusivity scores [0.523, (0.251, 0.812), BSR = 3.81, *p* < 0.001], with an *R*^2^ of 0.222 [95%CI: (0.065, 0.418)]. Thus, DTI parameters had moderately strong associations with insular network functional connectivity amongst the acutely concussed athletes. Neither the FA effects (BSR = −0.12, *p* = 0.904) nor the MD effects (BSR = −0.08, *p* = 0.938) were significantly influenced by adjusting for demographic covariates of age, sex, or HOC.

**Table 3:** White matter clusters with significant salience effects, indicating regions where fractional anisotropy (FA) and mean diffusivity (MD) are associated with appraisal mismatch network expression scores; the identified regions correspond to the brain maps shown in Figures 3A,C, respectively.

		**Brain region**	**Center of mass (MNI)**			**Cluster volume (mm^**3**^)**	**Peak BSR**
FA	1	Posterior thalamic radiation L	−38	−54	−2	192	−5.09
	2	Posterior thalamic radiation L	−36	−40	12	128	−5.42
MD	1	Posterior corona radiata R	30	−54	26	5,744	5.56
	2	Superior longitudinal fasciculus L	−30	−38	30	4,992	5.61
	3	Anterior corona radiata R	30	34	10	824	5.17
	4	Posterior thalamic radiation L	−40	−60	−2	592	5.02
	5	Posterior thalamic radiation L	−36	−74	8	296	4.67
	6	Body of corpus callosum R	4	2	28	296	3.84
	7	Posterior thalamic radiation L	−18	−90	−6	184	4.85
	8	Cerebral peduncle R	16	−10	−12	144	3.80
	9	Posterior thalamic radiation R	24	−90	2	144	4.08
	10	Superior longitudinal fasciculus L	−50	−44	38	144	4.77
	11	Superior corona radiata R	18	−4	58	112	3.82
	12	Superior corona radiata L	−18	−22	70	88	4.03

## Discussion

In concussion management, computerized cognitive testing is often a complimentary tool to symptom assessments, although weak correlations between performance on NCTs and the severity of self-reported symptoms are often reported (3–5). To date, the neural underpinnings of this discrepancy have not been well-studied. The present study used resting-state fMRI to examine this issue for the first time, focusing on the role of insular functional connectivity in the self-appraisal of cognitive function, during the acute phase of injury. This was examined using a flexible multivariate approach that simultaneously identified insular subregions and whole-brain networks where connectivity was related to clinical indices of symptoms and cognition. The primary study finding is that a discrepancy between the relative severity of self-reported cognitive symptoms and relative performance on an NCT battery (i.e., “appraisal mismatch”) is associated with insular connectivity to frontal and midline structures, as initially hypothesized, with insular-cingulate connections being most robustly identified. There is also evidence that these effects are related to concussion pathophysiology, as reduced FA and increased MD were seen in the white matter of concussed athletes with insular connectivity patterns related to symptom over-reporting.

Analysis of the main effects of concussion identified reductions in insular connectivity to a distributed set of brain regions, suggesting an overall disruptive effect of concussive impact on insular integration. The identified brain regions were primarily anterior medial, and included the orbitofrontal cortex, cingulate, and amygdala. These regions have extensive structural and functional connections with the insula (7, 50), but are also located in areas of high vulnerability to compressive forces during injury, caused by the brain impacting with the skull (51, 52). Given the role of these connections in interoception, emotion processing, and pain perception (53–58), such disruptions to insular connectivity likely contribute to the broad array of somatic, cognitive, and affective disturbances that are frequently reported after a concussion. In particular, the amygdala is implicated in automatic emotional responses, whereas the anterior insula is involved in the subjective experience of these emotion-states (7). Interestingly, the insular effect sizes tend to be highest in dorsal mid/anterior subregions, but all subregions show significant involvement. The widespread insular engagement is consistent with anatomical tracing, which shows substantial overlap of anatomical connectivity within neighboring insular divisions (59). Furthermore, the present results show insular functional connectivity to both posterior and anterior regions in the brain, which have anatomical connections to posterior and anterior insular subdivisions, respectively (60). Nevertheless, the ability to distinguish between insular subregions likely depends on many study variables (e.g., scan parameters, sample demographics, and processing pipeline), hence further research is needed to establish definitively whether the full insular cortex is consistently affected by concussion and implicated in cognitive self-appraisal afterwards.

Analysis of the composite measures of symptoms and cognition found no significant effects for overall severity score z(SYM)+z(COG). This is noteworthy, as studies of acute concussion have previously identified networks with insular involvement that are related to total symptom severity and speed of NCT performance (10–12). Despite these findings, the present results suggest a lack of common insular substrate for both perceived and actual cognitive deficits. By contrast, a significant association with mismatch score z(SYM)-z(COG) was observed, suggesting an insular substrate for mismatch in cognitive self-appraisal among the concussed athletes, as hypothesized. Moreover, insular connectivity was positively associated with the mismatch score. This is consistent with the role of the insula in self-monitoring and detection of salient stimuli (7, 50), and suggests that under-engagement of insular networks gives rise to the under-reporting of cognitive issues [i.e., z(SYM) < z(COG)], whereas over-engagement of these networks gives rise to the over-reporting of issues [i.e., z(SYM)>z(COG)]. Stated otherwise, these results indicate that accurate self-appraisal after concussion requires that insular functional connectivity be well-calibrated.

The regions showing appraisal-related connectivity to the insula included inferior, middle and superior frontal areas, which are structurally connected to the dorsal anterior insula (61). These findings are aligned with studies of MCI and AD, where frontal lesions and impaired function are correlated with anosognosia and deficits of self-perception (15–18). More generally, there is evidence that the frontal cortex plays a key role in representations of the self (62, 63), with the present results suggesting that integration with the insula influences the accuracy of this representation. Connectivity effects were also seen within sensorimotor nodes, including precentral, postcentral and paracentral areas, which are structurally connected to the mid-posterior insula (61). The insular-motor connections are likely detected because the present study focuses on speed of performance during NCTs. Similarly, insular-sensory connections may play a role in the detection of impaired speed of performance (i.e., captured in complaints such as “feeling slowed down”) (7). Intriguingly, connections between the insula and mid/anterior cingulate regions were also identified. They were also the sole connections that overlapped with the main effects of concussion, suggesting that these areas are most likely to have concussion-driven effects on self-appraisal. The high effect sizes in both anterior/mid insula and cingulate cortex are consistent with structural models of connectivity (61) that suggest insular-cingulate connections are congruent along the anterior-posterior axis. Midcingulate structures, in combination with the anterior insula, play a key role in cognitive control and integration with motor response (64), thus it is reasonable that they are implicated in self-appraisal of the speed of task performance. Similarly, midcingulate regions have been implicated in multisensory perception of bodily orientation in space (65), which may be critical for sensing of deficits in performance. This provides further evidence that perception of cognitive deficits may partly depend on self-perception of movement/orientation during action performance. As with the main effects analyses, insular effect sizes tended to be slightly higher in dorsal mid/anterior subregions, but all subregions showed significant involvement. The overall involvement of insula in self-appraisal is unsurprising, as a posterior-to-anterior remapping of interoceptive signals is thought to enable the perception of interoceptive information (66).

Diffusion imaging analyses also identified significant associations between insular network functional connectivity and white matter microstructure, among concussed athletes. Prior studies of concussed individuals, imaged within the 1st week post-injury, have reported decreases in FA and increases in MD within white matter tracts (67–70), which are often interpreted as a combination of microstructural injury, edema and neuroinflammatory response. This suggests that the appraisal of cognitive issues is tied to microstructural disturbances in the brain. Interestingly, disturbances in MD effects are considerably more spatially extensive than FA disturbances, which is consistent with milder forms of injury (41, 71) and suggests that the observed effects are mainly due to reversible change such as edema (72, 73) and neuroinflammation (74), rather than axonal injury. Thus, one potential interpretation is that patients with greater MD have a greater neuroinflammatory response, which may induce “sickness behavior,” including feelings of malaise, anxiety and altered cognition (75–77), all of which may lead to greater self-monitoring and subjective feelings of cognitive problems. Intriguingly, this would suggest that the psychological and behavioral effects of neuroinflammation play a role in cognitive self-appraisal after concussion. However, the contributions of axonal injury to the present DTI findings cannot be ruled out definitively (67), hence it is also possible that insular network hyper-connectivity is induced as a response to more extensive white matter damage. Advanced diffusion weighted imaging techniques such as neurite orientation dispersion and density imaging (NODDI) may provide more clarity about the observed microstructural effects (46, 78).

Although this study provided significant, novel insights into the mechanisms of cognitive self-appraisal after concussion, the findings should be considered in the context of limitations in the experimental design and its execution. In particular, the study examined associations between functional connectivity, symptoms and cognition cross-sectionally. The measure of appraisal mismatch therefore depends on the assumption that inter-individual variations in NCTs are predominantly concussion-related and not due to baseline cognitive differences. This is supported by evidence that baseline and normative NCT scores have comparable sensitivity (79, 80), and both cross-sectional measures of reaction time and change scores from baseline were previously found to have similar correlations with functional brain networks (12). A similar concern surrounds the symptom data, as the chosen measure of appraisal mismatch presumed that subject symptom reports are calibrated to a common scale, allowing a meaningful comparison across individuals. Although more difficult to ascertain, there is corroborating evidence that symptom severity predicts time until full clinical recovery (81, 82), and functional brain networks have been identified that are reliably correlated with acute symptom severity scores (11, 41). Another study limitation is that it is unclear whether the observed variations in insular connectivity were induced by concussion or represent more general “trait” variations between individuals—i.e., patients who were more prone to over-reporting of cognitive issues may have had higher pre-injury insular connectivity. As a final point of consideration, the present study focused on speed of task performance as a common deficit after concussion, which is broadly reflective of information processing speed (83). However, there may be other aspects of cognition (e.g., visual search, working memory, and cognitive control) with distinct neural substrates. To better understand these issues, there is a need for further prospective studies that include pre-injury baseline and acute assessments, with more detailed measurement of different aspects of symptoms, cognition, and brain function.

Given the importance placed on both symptom assessments and NCTs in concussion assessment, it is critical to better understand the cause of errors in self-appraisal after injury. Although there is a substantial body of research on these issues for more severe neuropathologies such as stroke, AD, MCI, and TBI, little is known in milder cases of brain injury, such as concussion. As the etiology of neurocognitive deficits is distinct from those present in other neurological populations, and that the deficits tend to be subtler in nature, this provides a unique opportunity to gain novel insights into issues of accuracy in the self-appraisal of cognitive deficits. The present study suggests that the insula plays a key role in self-appraisal, which is consistent with literature evidence that it is an integration hub involved in interoception, self-monitoring, and cognition. These findings have significant clinical relevance, by providing an increased understanding of the mechanisms by which self-appraisal is affected after concussion. This is relevant for establishing better integrated approaches to assess severity of injury and recovery, with applications in determining safe return to sport, work and daily activities. The identified clinical and neural measures also provide a potential framework for interrogating mechanisms of long-lasting complaints in the absence of overt cognitive deficits.

## Data Availability Statement

The datasets presented in this study can be found in online repositories. The names of the repository/repositories and accession number(s) can be found at: The datasets analyzed for this study can be found in the figshare repository at https://figshare.com/s/d758e5e696332f8b3edf.

## Ethics Statement

The studies involving human participants were reviewed and approved by University of Toronto and St. Michael's Hospital research ethics boards. The patients/participants provided their written informed consent to participate in this study.

## Author Contributions

NC was involved in study design, analysis planning and execution, interpretation of results, and manuscript writing. MH and SG were involved in study design and critical revision of manuscript. TS was involved in study design, interpretation of results, and critical revision of manuscript. All authors contributed to the article and approved the submitted version.

## Conflict of Interest

The authors declare that the research was conducted in the absence of any commercial or financial relationships that could be construed as a potential conflict of interest.
