# Supplementary material for: Insular Connectivity Is Associated With Self-Appraisal of Cognitive Function After a Concussion
Source: Front Neurol. 2021 May 21;12:653442. doi: 10.3389/fneur.2021.653442 (PMC8175663; doi:10.3389/fneur.2021.653442)
Supplement: Supplementary file 1 [file Data_Sheet_1.docx]

**Supplemental File 1:** Athlete numbers by sport

**Table S1:** athlete numbers by sport, for both male (M) and female (F) groups, for N=136 controls and N=59 concussed athletes.

| **CONTROL** | **CONCUSSION** |
| --- | --- |
| Squash (1M)  Water polo (1M)  Lacrosse (7M* / 6F)  Basketball (3M / 8F)  Rugby (6M* / 4F*)  Football (9M*)  Soccer (14M / 8F)  Hockey (23M* / 29F)  Volleyball (5M / 12F) | Mountain biking (1M/1F)  Softball (1F)  Figure skating (1F)  Rowing (1F)  Swimming (1F)  Water polo (3F)  Lacrosse (1M / 1F)  Basketball (2M)  Rugby (10M* / 10F*)  Football (8M*)  --  Hockey (6M* / 10F)  Volleyball (2M) |

* collision sports, defined as involving routine, purposeful body-to-body contact^1^

1. Meehan III, W.P., Taylor, A.M., Berkner, P., Sandstrom, N.J., Peluso, M.W., Kurtz, M.M., Pascual-Leone, A. and Mannix, R. (2016). Division III collision sports are not associated with neurobehavioral quality of life. Journal of neurotrauma 33, 254-259.
