# Supplementary material for: Insular Connectivity Is Associated With Self-Appraisal of Cognitive Function After a Concussion
Source: Front Neurol. 2021 May 21;12:653442. doi: 10.3389/fneur.2021.653442 (PMC8175663; doi:10.3389/fneur.2021.653442)
Supplement: Supplementary file 2 [file Data_Sheet_2.docx]

**Supplemental File 2:** Multivariate outlier detection

To identify outlying imaging data, we used a simple protocol that quantified overall deviation of brain maps from a robust mean map. For a parameter map of interest (functional connectivity, fractional anisotropy, mean diffusivity), the data of subject *s* = 1…*S* are formed into vectors $\boldsymbol{x}_{s}$ consisting of *V* measurements. All subject data are then concatenated into a 2D matrix $\boldsymbol{X}$ of dimensions (*V* x *S*). We then compute map of median values $\boldsymbol{x}_{med}$ over all subjects, then obtain for each individual the sum-of-squares deviation $SSD\left( s \right)=\sum_{v=1}^{V} \left( \boldsymbol{x}_{s}\boldsymbol{-}\boldsymbol{x}_{med} \right)^{2}$. We then fit a gamma distribution to the set of SSD values using maximum likelihood estimation, and subsequently obtain p-values as the reciprocal of the cumulative probability for each participant’s SSD. Finally, we identify all datapoints having significant SSD values at a False Discovery Rate (FDR) threshold of 0.05.

Figure S1 below shows an example plot from the insular connectivity data, where each vector $\boldsymbol{x}_{s}$ corresponds to the set of 12x246=2,952 connectivity values. For connectivity, only a N=1 participant showed abnormal connectivity values at an FDR of 0.05. For DTI measures of FA and MD, $\boldsymbol{x}_{s}$ corresponds to the map of 56,037 white matter voxels. We identified one control that was an outlier in FA alone and one that was an outlier in both FA and MD; we also identified one concussed athlete that was an outlier in MD alone. Thus, a total of N=3 participants were excluded.


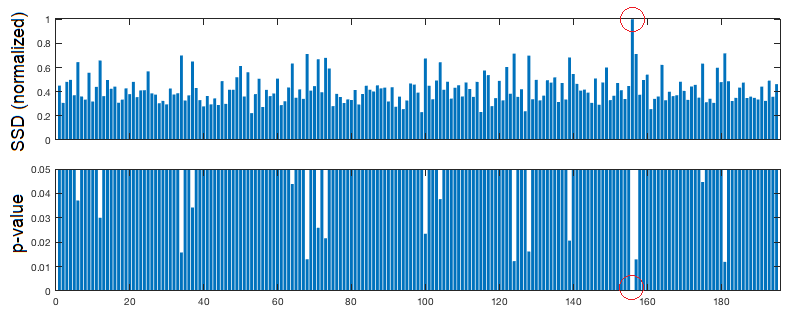


**Figure S1**: plot depicting outlier estimation, including (top panel) sum-of-squares deviations (SSD) from the median brain map, with values rescaled to the range of [0, 1]; and (bottom panel) corresponding p-values, focusing on the interval of [0, 0.05].
